# The Younger Alexander

**Published:** 1861-08

**Authors:** 


### HALL'S JOURNAL OF HEALTH.

Our Legitimate Scope is almost boundless: for whatever begets pleasurable and harmless feelings, promotes Health; and whatever induces disagreeable sensations, engenders Disease.

WE AIM TO SHOW HOW DISEASE MAY BE AVOIDED, AND THAT IT IS BEST, WHEN SICKNESS COMES, TO TAKE NO MEDICINE WITHOUT CONSULTING A PHYSICIAN.

Vol. VIII.] AUGUST, 1861. [No. 8.

### THE YOUNGER ALEXANDER.

When Archibald Alexander died, it was felt to be a loss to the whole Presbyterian Church, which could not be fully repaired; he had no second in his sphere, and now that his son James W. has so soon followed him, the same feeling prevails, and his people especially are deeply sensible of the fact that their loss can never be supplied, that his place will never be filled as he filled it.

We were among the privileged attendants on his preaching, and an infirmity of one of the members of our household requiring a seat nearest the pulpit, opportunities of observation and hearing were afforded, which are the more highly valued, now that they are forever gone.

Dr. James W. Alexander was a man who seemed to do everything at the right time, at the right place, and in the right manner. He was the most considerate of men. He appeared to be able to put himself in place of others, and to act just as they would have him do, as to all that was right. His heart was always overflowing with a wish to do everybody good. If he could not do a desired thing, he was able to show it so convincingly that the applicant felt at once as if he no longer wanted him to do it. With his ever present willingness to do a good turn for others, the smallest item of attention or good will towards himself, wakened up the most lively expressions of obligation, as if his abiding feeling were that he was unworthy of anything from anybody, when at the same time he was working out his life in the service of others. His heart seemed to be always going up to heaven. When he was a hearer instead of a speaker, his favorite position was to have a hand resting towards the knee. We could always tell by the motion of that hand, now cold in death, whether there was anything strikingly devotional in the person officiating, either as to the sermon or prayer. In the reading of the hymns by others it was the same thing; he seemed to find constant causes of ejaculation to the Father of us all, whether in hymn, or sermon, or prayer, or speech, as if his heart was always with God, as if it could extract sweeter than angel's food from commonest things.

It is very likely that no people ever had towards their pastor so large a feeling of affectionate reverence; and never did a people have a more ceaseless, tearful, and tender solicitude for their welfare and happiness exercised towards them on the part of their minister.

Not a man of them all will cease to remember his calm and quiet and unpresumptuous demeanor, as at the instant of the first note of the organ they were accustomed to turn their eye to the sacristy and note his ascent to the pulpit, so reverential, so all absorbed, as if he was whelmed with a sense of his responsibility.

He had no airs. It was impossible for him to do anything for mere effect. He never framed a grandiloquent sentence in our seven years' experience. Holy, humble fervor were the weapons of his warfare.

Dr. Hodge, of Princeton, describes his sermons as being often "a string of pearls." Never was there a more appropriate expression; as proof, see notes of a sermon taken verbatim during the crisis of '57, in the February, '58, number of *Hall's Journal of Health*, under the title of "Careworn."

Of all the sentiments expressed by him in the course of years, that which is most distinct, whether rendered so by its unexpectedness, or by the pains which he took to prevent his hearers from being surprised by it, was this: that the longer we lived, the severer would be our trials. He spoke this with an earnestness and solemnity which implied not only a uniform observation but a steady individual and personal experience; this was several times presented in the course of a few months.

"The Bible is the poor man's friend," was another sentiment, repeated with a power of utterance, in connection with the subject under discussion, peculiar to himself.

In speaking, on one occasion, of the value of the Scriptures in the application of their principles in successfully conducting the affairs of life, giving as an example the stern faith and general thrift of

Scotch Presbyterians, whose distinctive characteristic it was to teach their children the Scriptures which inculcated rules of conduct applicable to all times and circumstances, he repeated, "He that hateth suretyship is sure." The wisdom of the lesson was felt by every hearer.

We have often contemplated with admiration, because of its lovingness and sincerity, the little gathering that would take place in front of the pulpit after each discourse, the meeting of the elders with their faithful and endeared minister, as if they said in the face of the whole assembly, "We'll stand by you in all that you have said to-day." One by one they would come up with their friendly and respectful smile, Halstead, and Irvin, and Smith, and Walker; for a while there was another, who, in his great old age, seemed to receive something comforting every time he took his pastor's hand: it was old Mr. Auchincloss; but he is dead years ago; they have joined hands again in the upper sanctuary, and "they shall go no more out." But there was a more aged pilgrim still than they all, "and she was a widow," and yet surviving near her nineties. The Doctor never failed to meet Mrs. Bethune (the mother of a worthy and distinguished son) half way, and taking her time-worn hand in both of his, he seemed to feel as if he were the honored one, in being permitted thus to greet an aged saint on earth, who, any day, might be called to "go up higher." There was a respectfulness and an affectionateness in his manner towards her which were well calculated to make her take her trembling yet eager steps, to get his greeting every time she was permitted to come up to the great congregation.

Dr. Alexander was a man of liberal sentiments and a wide heart. In private and in public he has expressed his admiration of certain sentiments and practices of the Society of Friends. He had been thrown among them in early life, and seemed to speak with pleasure of the associations of the long departed past.

An Episcopalian mother expressed a wish that Dr. Alexander should christen her children. "No," said he, "I know your minister well, and respect and love him; he will take good care of them, and I would not have him think that I interfered with his lambs."

Shortly before he died, one of his own members, who, for nearness, had sent her small children to an Episcopal Sunday-school, asked him if she should continue to do so. "By all means for the present; Dr. Tyng is my personal friend; it is impossible for me to make my Sabbath-school as interesting as he does; let them remain."

The admiration with which his people regarded his preaching, may be inferred from the fact that there was a uniform and general feeling of disappointment the moment any other person appeared in the pulpit; and we could frequently foretell that he was not to preach, on the moment of entering the church, by the comparative thinness of the audience, for, some how or other, the more inquisitive would find out when he was not to preach. His was perhaps the most uniformly filled church in New York. As a general thing, it was difficult to find a vacant seat for a stranger, in the afternoon. For more than a year before his death the lecture room on Thursday nights was crowded; it held over four hundred persons, and chairs had to be brought in besides. We speak from observation, for we were seldom absent. His kindness of heart was such that he was uneasy if he saw a single person standing. We have seen him hand the only chair left in his desk, for some stranger or aged person.

His humility and ungraspingness were strikingly exhibited in the year of the panic, in his persistent refusal to permit his salary to be increased by a thousand dollars, saying that for his habits of life his present pay was sufficient. But, without his knowledge, his considerate session invested the amount, to which a faithful people has promptly added enough to make the sum of twenty-five thousand dollars, to place his bereaved family in comfortable circumstances as long as they live. They have done it out of love for him. "What a people and how unworthy am I of such demonstrations" would be his exclamation in the flesh. We sometimes feel as if we would like for him to know it, for it is too soon yet for us to "make him dead."

If asked what was the most distinguished thing about him, we would most unhesitatingly say it was his prayers. Of all we have ever heard uttered, or read of the uninspired, Dr. Alexander's were the most devotional, the most heavenly. There was no human condition they did not reach. Sabbath after Sabbath for more than six months before his death, we debated whether we should not take down his prayers verbatim. If it could have been done without his observation or knowledge, we certainly should have done it. We next concluded to hire a reporter, but the difficulty in getting a faithful and conscientious one seemed to be insuperable in New York. His death found us inquiring for one. He seemed to get right at once into the presence of his Maker, and as if wanting to improve his opportunity before he got away, his great broad heart would take all humanity within its folds.

He seemed familiar with every phase of human sorrow. In a single prayer, and we made note of it at the time, he petitioned for those who were kept from the house of God by inclement weather, by the sickness of themselves or near relations, by the compulsion of others; for those who were suffering in their good name in person, or in the person of others; for those who were in actual want of food or raiment; for those who were anticipating revealments which would affect their social position; for those who were made bankrupt; for those who were anticipating the loss of fortune; for those who were writhing under the apprehension of failure to meet maturing pecuniary obligations; for those who were hardened by worldly entanglements; for those whose hearts were wrung by the mental derangement of friends, or of their own families; for those who were afraid they should themselves go mad; for those who felt they were castaways from God, and believed their perdition sealed. The impression made on our mind was so strong at the time, we felt almost ready to exclaim audibly, "What a miserable congregation this is!" His prayers were uniformly most impressive. Forgetting himself and his congregation, he would carry away at times in his great warm heart the wants of a world, and lay them right down at the mercy seat for God to look at, and pity and deliver.

The influence which Dr. Alexander possessed over his people, without their feeling it, and in a certain sense, without his exercising it, may be inferred from the liberality of his congregation. On the first Sabbath morning of each month he was accustomed to preach a sermon in reference to one of the leading benevolences of the times, when checks would be thrown into the plate for five hundred, a thousand, and three thousand dollars. The amount of a morning's contributions was from three to seven thousand dollars. The rapidity with which his influence and power grew in this regard, may be inferred from the fact, that in 1852, when he took charge of the Fifth Avenue Church, the contributions were less than four thousand dollars, while for several years past they have averaged over sixty thousand, besides many thousand more of private and unacknowledged charities. His membership more than doubled in seven years, numbering now over seven hundred. These are some of the tangible evidences that he lived to purpose; and now that he is gone, at the early age of fifty-four, the waves of his influence will go out from his writings, benefiting and blessing wherever they roll, and many yet unborn will have reason to bless God throughout the ages that such a grand heart ever lived.

Thus wrote we near two years ago, for the FIRESIDE MONTHLY, and reproduce it here to impress an important lesson in hygiene, by inquiring: Why did he die so soon? There are clergymen a score of years older than he was, who are still powerful and efficient workers in the great field of the world. It seems to us that an uncomputed wealth of influence for good was lost to the Church and to religion in general by his having passed away in the very meridian of his days. We can not say that it was by God's appointment; at least we can not feel fully satisfied with resting in such a conclusion. It is safer and wiser to say that three-score years and ten should be the average measure of our days, and that those who leave the stage sooner do so in consequence of their own conduct or that of their fellow-men; the uncontrollable agencies of the "elements" excepted.

Any one—the feeblest—can commit an error; it requires a MAN to frankly acknowledge it. There is a greater courage than that of marching right in the face of belching cannon in the frenzy of battle; it is that of enduring the agonies of the wheel and the stake for hours together, when a single word would cease the torment instantly. Only great minds and heroic hearts are capable of deeds like these. Last month a great name was mentioned who endured hunger in uncomplaining gentleness for two years. Within a dozen hours the common herd becomes fretful, passionate, and impatient of hunger. Not less great was the author of the *Cause and Cure*, than was the subject of this article, who, like too many Virginians, became extravagantly addicted to the use of tobacco, so much so that before he was thirty, it threatened his intellect, and that too before he became aware of the fact that it was owing to this species of intemperance that both mind and body were failing together. But no sooner was it distinctly placed before him, than by one heroic resolve he shattered the manacles which bound him, and never after took another "chew." But it was not done soon enough to save him from life-long sufferings. For years before his death, the palsied shaking of his head was apparent to all who heard him, while he was only kept out of the grave by frequent release from official duties and the recreations of travel. He repeated it to the writer, and had no hesitation in stating it to his friends, that his bodily infirmities were laid in the extravagant use of tobacco in his youth; it robbed him of twenty years of life and of honorable usefulness to the church of his choice. Need another word be said to induce any young gentleman who is preparing for professional life and who is a slave to the weed, to rise in the might of his manhood and say: "I will never use it again?"

Tobacco in any form is not only a narcotic but it is a stimulant also; it not only blunts the sensibilities, but it goads both mind and body to unnatural activities, and the machine made to run faster than was ever intended, wears out so much the sooner and long before its time, and stops forever! "Doctor, why do you use tobacco so?" said we a few months since to a physician whom we met on the street, whose whole mouth seemed to be so full of it that he was crunching it as persons do who have a mouthful of water-melon. "I must do it to keep down the pain in my teeth." We never saw him afterwards, and the record of his death reads thus in the *American Medical Times*: "He suffered from disease of the aortic valves of the heart, leading to dropsical effusion, resulting in mortification of the legs and feet, ending in tetanic symptoms and death." What a fearful concatenation of human maladies: heart disease, dropsy, mortification, and lockjaw! any one of which ailments is enough to destroy an iron frame. But note: the disease began in the heart, that heart which had been kept in excess of excitement for so many years by the long, steady, and large use of tobacco.

With beacon-lights like these shining full in his eyes, the man who persists in the employment of tobacco in any shape or form, and who, to all arguments against its employment, can only reply, "I can't," or "I won't," only confesses himself a moral impotent or a reckless criminal; for that it is a crime to knowingly persist in practices which are destructive to the body, can scarcely be denied.

Tobacco does relieve pain, but it never cures, never removes, never eradicates pain; it only blunts the sensibilities. Pain is nature's warning that something wrong is going on in the system, and urges its rectification; tobacco suppresses the cry, by rendering the parts insensible to hurtful agencies, but those agencies do not cease, and as incessantly as before work away at the demolition of the body: a burning building is not the less in course of destruction because the inmates do not see or feel the fire. But tobacco excites; it stimulates to exertion which would not otherwise have been made. All exertion is at the expense of vital force, of life-power, of nervous energy, and in proportion as these are drawn upon in advance, a time must come, as with a balance in bank, when there are no assets to be drawn upon, and the life-power is bankrupt, the body fails and passes into the grave. Thus it is that when persons come to their final sickness, who have used stimulants largely, whether of tobacco, opium, or spirits, there is a lack of recuperative power; their disease is of the typhoid type; there is no elasticity of mind or body; the latter is weak, the former is asleep, and the patient lies for hours and days in an insensible state, or is only made conscious by shaking the body violently, by loud words, or by some acute pain, the death-throe of nature for existence. Mr. Webster died in this way, so did Mr. Douglas, and Count Cavour, and Dr. Rease, and multitudes of other eminent men, who by keeping the system stimulated beyond its natural condition, exhausted its vitality, its nervous power, in advance; hence, when serious illness came, there was nothing to fall back upon, no recuperative power, and they now sleep in the grave! Webster and Douglas used alcohol; Choate used opium, as was said; Reese used tobacco; Cavour was a gourmand, exhausted the life-power in advance, by overtaxing the powers of the stomach. It is notorious that the men who, working about the breweries of London, swill beer by the gallon daily, do, by the time they reach forty years, become so deficient in recuperative power that an abrasion of the skin, a cut of the finger, and even the puncture of a splinter or the scratch of a pin, is almost as certainly fatal as a bullet through the brain or body. These are terrible teachings, but they are true.

